# Machine learning analysis of SERS fingerprinting for the rapid determination of *Mycobacterium tuberculosis* infection and drug resistance

**DOI:** 10.1016/j.csbj.2022.09.031

**Published:** 2022-09-26

**Authors:** Liang Wang, Xue-Di Zhang, Jia-Wei Tang, Zhang-Wen Ma, Muhammad Usman, Qing-Hua Liu, Chang-Yu Wu, Fen Li, Zuo-Bin Zhu, Bing Gu

**Affiliations:** a Laboratory Medicine, Guangdong Provincial People’s Hospital, Guangdong Academy of Medical Sciences, Guangzhou, Guangdong Province, China; b Department of Laboratory Medicine, Medical Technology School, Xuzhou Medical University, Xuzhou, Jiangsu Province, China; c The Affiliated Infection Diseases Hospital of Xuzhou Medical University, Xuzhou, Jiangsu Province, China; d Department of Bioinformatics, School of Medical Informatics and Engineering, Xuzhou Medical University, Xuzhou, Jiangsu Province, China; e Jiangsu Key Laboratory of New Drug Research and Clinical Pharmacy, School of Pharmacy, Xuzhou Medical University, Xuzhou, Jiangsu Province, China; f State Key Laboratory of Quality Research in Chinese Medicines, Macau University of Science and Technology, Taipa, China; g Department of Biomedical Engineering, School of Medical Imaging, Xuzhou Medical University, Xuzhou, Jiangsu Province, China; h Laboratory Medicine, The Fifth People's Hospital of Huai'an, Jiangsu Province, China; i Department of Genetics, School of Life Sciences, Xuzhou Medical University, Xuzhou, Jiangsu Province, China

**Keywords:** *Mycobacterium tuberculosis*, Infectious disease, Antibiotic resistance, Raman spectrometer, Machine learning, Deep learning

## Abstract

Over the past decades, conventional methods and molecular assays have been developed for the detection of tuberculosis (TB). However, these techniques suffer limitations in the identification of *Mycobacterium tuberculosis* (*Mtb*), such as long turnaround time and low detection sensitivity, etc., not even mentioning the difficulty in discriminating antibiotics-resistant *Mtb* strains that cause great challenges in TB treatment and prevention. Thus, techniques with easy implementation for rapid diagnosis of *Mtb* infection are in high demand for routine TB diagnosis. Due to the label-free, low-cost and non-invasive features, surface enhanced Raman spectroscopy (SERS) has been extensively investigated for its potential in bacterial pathogen identification. However, at current stage, few studies have recruited handheld Raman spectrometer to discriminate sputum samples with or without *Mtb*, separate pulmonary *Mtb* strains from extra-pulmonary *Mtb* strains, or profile *Mtb* strains with different antibiotic resistance characteristics. In this study, we recruited a set of supervised machine learning algorithms to dissect different SERS spectra generated via a handheld Raman spectrometer with a focus on deep learning algorithms, through which sputum samples with or without *Mtb* strains were successfully differentiated (5-fold cross-validation accuracy = 94.32%). Meanwhile, *Mtb* strains isolated from pulmonary and extra-pulmonary samples were effectively separated (5-fold cross-validation accuracy = 99.86%). Moreover, *Mtb* strains with different drug-resistant profiles were also competently distinguished (5-fold cross-validation accuracy = 99.59%). Taken together, we concluded that, with the assistance of deep learning algorithms, handheld Raman spectrometer has a high application potential for rapid point-of-care diagnosis of *Mtb* infections in future.

## Highlights

• Handheld Raman spectrometer is able to generate SERS spectra with sufficient quality for *Mycobacterium tuberculosis* detection.
• It is feasible to accurately discriminate *Mtb*-positive sputum from *Mtb*-negative sputum through SERS spectrometry.
• Pulmonary and extra-pulmonary *Mtb* strains were able to be accurately distinguished via SERS spectral analysis.
• Profiling of antibiotic resistance of *Mtb* strains was successfully achieved through machine learning analysis of SERS spectra.

## Graphical abstract

**Figure:**
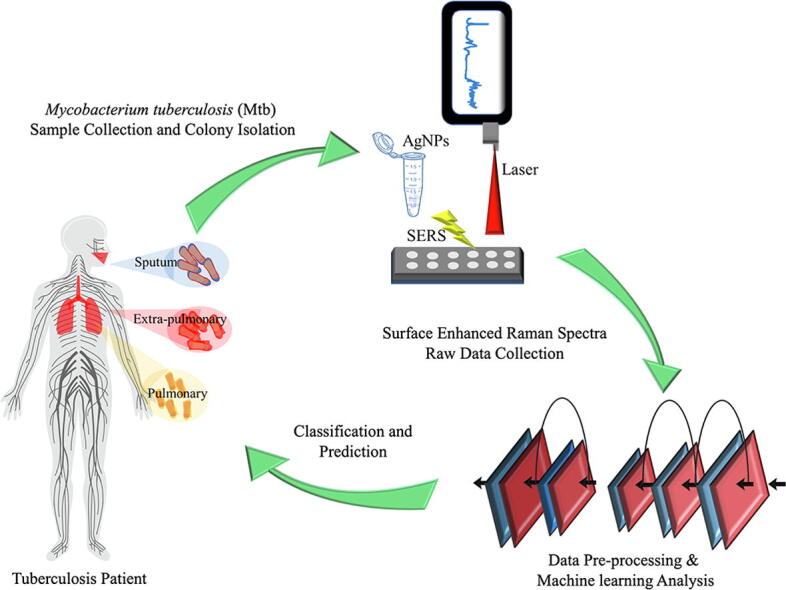

## 1 Introduction

Tuberculosis (TB) is an ancient and severe infectious disease that is caused by the highly contagious airborne bacterial pathogen *Mycobacterium tuberculosis* (*Mtb*), which mainly attacks lungs and is often terms as pulmonary tuberculosis (PTB) [1]. In addition, TB can sometimes cause damages to other body parts such as brain, kidneys and the spine, which is often known as extrapulmonary tuberculosis (EPTB) and accounts for about 20% of all TB cases [1]. Since 1997, World Health Organization (WHO) has been publishing annual global tuberculosis report to evaluate global TB situation and assessing developments in prevention, diagnosis, and therapy of *Mtb* infections at country, regional and global levels [2]. According to the latest global epidemiology of tuberculosis by Global Tuberculosis 2021 Report [3], a total of 10 million people has been estimated to fall ill with TB while 1.5 million people died from TB in 2020, which reversed global progress in tuberculosis control in over a decade and represented the first year-over-year increase in tuberculosis deaths since 2005 [4]. Currently, it is estimated that around 2 billion people are infected with *Mtb* [5], which makes TB the leading fatal single infectious disease worldwide [6]. Globally, the severity of TB epidemics differs among countries and regions while China ranked the second among the top 30 high-burden TB countries just behind India, which accounted for 9% of all the TB cases [7] and indicated that China was still far from achieving the targets set out in the WHO End TB Strategy [8]. Thus, it is important to strengthen the national health policy and improve capacities of TB screening, diagnosis and outpatient treatment so as to prevent, control and finally terminate TB infections in China.

*Mtb* belongs to the family of *Mycobacteriaceae* and is one of the oldest known pathogenic bacteria in human beings that was first discovered in 1882 by Robert Koch [9]. However, it belongs to neither Gram-negative nor Gram-positive bacterium due to its waxy cell envelope, a specialized dual membrane structure mainly formed by mycolic acids (MAs) covalently linked to the polysaccharide cell wall and free lipids that leads to the bacterial resilience and infectivity [10]. After *Mtb* infection, infected individuals could be generally classified into two categories: latent tuberculosis infection (LTBI), also known as the asymptomatic clinical state with no transmission risk (≈ 90%), and active TB disease with clinical symptoms such as cough, fever and night sweats (≈10%), though the binary view has recently changed and *Mtb* infection is considered as a spectrum of disease states [5], [11]. In certain circumstances, *Mtb* could establish an infection outside lungs, leading to the development of extrapulmonary TB (≈15% of infected cases) [12]. As for the treatment of tuberculosis, the core regimen requires a long-term antituberculosis therapy phase (6–9 months) through various combinations of four first-line drugs, that is, rifampicin, isoniazid, pyrazinamide and ethambutol [13]. However, late diagnosis will normally compromise the treatment effect. In addition, inappropriate administration of drugs and nonadherence to drug regimen will also cause the emergence of drug-resistant TB strains, leading to greatly enhanced risks and significant challenge to TB control. Thus, rapid diagnosis of *Mtb* species and accurate discrimination of drug-resistant and drug-sensitive *Mtb* strains would greatly facilitate the control and therapy of tuberculosis disease.

So far, multiple techniques have been developed for the fast and accurate identification of *Mtb* infections, which involve molecular methods, immunological assays, and direct culture and observations and so forth [12]. Common diagnostic tests include bacterial culture, smear microscopy, serological test and chest X-ray screening [14]. However, these methods suffer either insufficient testing inaccuracy or long turnaround testing time, not even mentioning the inconvenient applications in remote and rural areas [12]. For example, diagnosis relying on the culture of *Mycobacteria* requires high-level and expensive technological facilities and the process might take up to 2 months, causing significant delay of active tuberculosis diagnosis [15]. As for smear microscopy, although it is a rapid and cheap method for detecting *Mtb*, the method is less sensitive due to the requirement of more than 5,000 bacilli per mL of sputum for positive results [16]. In addition, smear microscopy is not specific for *Mtb* infection, is insufficient to distinguish between viable and dead cells, and incapable of detecting drug resistance [15]. Serological tests of *Mtb* infection are mainly based on the detection of antibodies and have been commercially available for many years; however, due to its inaccuracy for both pulmonary and extra-pulmonary TB diagnosis [17],World Health Organization (WHO) strongly recommended against the use of commercial serological tests for the diagnosis of TB disease [18]. As for radiology in the diagnosis of *Mtb* infection, radiograph contributes to risk stratification in terms of latent infection, previous inactive disease and active disease [19], which also falls into WHO recommendations as an adjuvant test in smear-negative tuberculosis (TB) diagnosis [20]. However, its efficacy is relatively low and interpretation of the results suffer the effects of human errors, which could be improved through computational algorithms and the use of artificial intelligence (AI) [12].

Therefore, novel methods and technological advancements are eagerly in need to achieve more rapid, less expensive, and more accurate results in order to defeat the highly contagious disease. Recent developments of *Mtb* diagnosis include digital droplet PCR (ddPCR), CRISPR, next-generation sequencing (NGS), microRNA detection, eNose, Raman spectroscopy (RS), AI processing, and graphene-based biosensors and so forth, among which RS, especially surface enhanced Raman spectroscopy (SERS), is a highly potential point-of-care technique (PoCT) that utilizes the effect of Raman scattering to detect and discriminate the unique molecular fingerprints of various bacteria [12]. Due to the complexity of SERS spectral data, classical statistical analysis is insufficient in the analysis of Raman spectra, while machine learning algorithms, including certain deep learning algorithms, are more appropriate for rapidly and accurately processing these data [21]. However, few studies compared the effectiveness of different machine learning algorithms in *Mtb* identification and discrimination via SERS technique.

In this study, we used a handheld Raman spectrometer (Anton Paar Shanghai Trading Co., ltd., China) to detect all the *Mtb* strains and clinical sputum samples and generate SERS spectra, which were then analyzed by six supervised machine learning algorithms, that is, CNN (Convolutional Neural Network), GRU (Gate Recurrent Units), LSTM (Long Short-Term Memory), MLP (Multilayer Perceptron), Random Forest (RF), and Support-Vector Machine (SVM). In particular, SERS spectra for culture-negative sputum samples (n = 4) and culture-positive sputum samples (n = 4) were first generated and then analyzed computationally, according to which the deep learning algorithm convolutional neural network (CNN) performed the best than other supervised machine learning methods (fivefold cross-validation accuracy 94.32%). Then, SERS spectra for pulmonary *Mtb* strains (n = 47) and extra-pulmonary *Mtb* strains (n = 11) were obtained, analysis of which via machine learning algorithms showed that CNN also had the highest fivefold cross-validation accuracy (99.86%). After that, *Mtb* strains with different profiles of drug resistance were explored via SERS technique. All the machine learning algorithms had good performed on discriminating *Mtb* strains with different drug-resistance profiles, among which CNN topped all other algorithms with fivefold cross-validation accuracy at 99.86%. Taken together, we concluded that SERS combined with deep learning algorithms could contribute to the rapid and accurate identification of *Mtb* strains in a variety of situations, confirming the promising future of SERS technique in *Mtb* diagnosis with the assistance of deep learning algorithms in clinical settings.

## 2 Methods and materials

### 2.1 Collection of clinical and bacterial samples

Both *Mtb*-negative and *Mtb*-positive sputum samples were collected from the Affiliated Infectious Diseases Hospital of Xuzhou Medical University and confirmed through smear microscopy (also known as acid-fast bacilli testing, AFB test). All the *Mtb* strains were isolated from the processed specimen of TB patients: pulmonary (sputum and bronchoalveolar lavage fluid) and extra-pulmonary (urine, pus, hydroperitoneum) samples, which were then inoculated into modified Lowenstein-Jensen (L-J) medium (BaSO Biotech Co., Zhuhai, China) for *Mtb* culture at the medical laboratory of the Affiliated Infectious Diseases Hospital of Xuzhou Medical University. Meanwhile, *Mtb* strains was confirmed through the performance of p-Nitrobenzoic acid (PNB)/Thiophen-2-Carboxylic acid Hydrazide (TCH) assay. All the cultures were incubated at 37 °C for 4 weeks. Antibiotic resistance and susceptibility of all *Mtb* strains were determined by Roche^TM^ Proportion Method and PCR-Reverse Dot Hybridization for Resistance Gene Mutation Detection. A total of 136 *Mtb* strains were included in this study. In particular, for all the pulmonary *Mtb* strains, 47 strains were rifampicin (RFP, R)- and isoniazid (INH, H)-susceptible (R-, H-), 9 strains RFP-resistant and INH-susceptible (R+, H-), 20 strains RFP-susceptible and INH-resistant (R-, H + ), and 47 strain RFP- and INH-resistant (R+, H + ). For details, please see **Supplementary** Table S1. As for extrapulmonary TB analysis, 11 *Mtb* strains (RFP+, INH + ) were included in this study. For details, please see **Supplementary** Table S2. Finally, for the direct analysis of clinical samples, 4 smear-positive and 4 smear-negative sputum samples were collected and investigated in this study. For details, please see **Supplementary** Table S3. The sputum was pre-treated with the equal volume of 4% (w/v) sodium hydroxide (NaOH) for homogenization via 20-min vortexing, which was then adjusted to pH = 7 with phosphate-buffered saline (PBS) and centrifuged at 3000 × g for 20 min. Discard the supernatant and mix the precipitate with 0.5 mL PBS for later use. As for *Mtb* colonies, all of them were isolated from clinical samples via culture and then were stored at −80 °C for later use. All the microbiological experiments involving *Mtb* strains were conducted in a licensed *P*3 Biosafety Laboratory by a certified clinical microbiologist (Xue-Di Zhang, co-first author of this study). Information of all patients concerning the sputum samples and *Mtb* strains was de-identified throughout the study to avoid any ethical issues.

### 2.2 Synthesis of silver nanoparticle (AgNPs)

AgNPs synthesis followed the routine procedures that were previously reported by Tang et al. with modifications [22]. Briefly, 33.72 mg of Silver Nitrate AgNO_3_ (Sinopharm, Beijing, China) was dissolved into 200 mL ultra-pure water (deionized distilled water, ddH_2_O) with heating via a benchtop magnetic stirrer (ZNCL-BS230, Shi-Ji-Hua-Ke Pty. ltd., Beijing, China) until boiling. After that, stop heating, add 8 mL of sodium citrate (1% wt) to the solution with stirring speed at 650 RPM until the mixture was cooled down to room temperature. Final volume of the solution was adjusted to 200 mL via ddH_2_O. 1 mL of the solution was transferred to a 1.5 mL Eppendorf tube, which was then centrifuged at 7,000 RPM for 7 min (Centrifuge 5430 R, Eppendorf, USA). After centrifugation, discard the supernatant and resuspend the pellet with 100 μL of ddH2O, which was considered as AgNPs substrate and was stored in the dark at room temperature for later use.

### 2.3 Measurement of SERS spectra

A loop of a single *Mtb* strain that was stored at 4 °C for short-term [3], [4], [5], [6] on solid agar medium was inoculated onto Roche^TM^ L-J medium and cultured in an aerobic incubator at 37 ± 1 °C for 4 weeks. Colonies in the medium were scraped and transferred into an Eppendorf (EP) tube containing 0.5 mL of sterile saline water and glass beads, which were then vortexed to disrupt *Mtb* clumps and generate homogenate cell suspension. 15 μL of previously prepared AgNPs was mixed with 15 μL of the homogenate *Mtb* solution through pipetting, which were then dropped onto the single crystal silicon wafer (P-type, Lijing Silicon Material Co., ltd., Zhejiang, China) to form a round spot, and dried naturally in biological safety cabinets (BSC). For the sputum sample, 15 μL of the homogenate sputum sample was mixed with 15 μL of AgNPs through pipetting, which were also dropped onto silicon wafer and dried naturally for detection purpose. All the Raman spectral data were acquired through an advanced Handheld Raman Spectrometer CORA100 (Anton Paar Shanghai Trading Co., ltd., China) in BSC within a certified *P*3 Laboratory at the Affiliated Infectious Diseases Hospital of Xuzhou Medical University. Parameters for Raman spectrometer were set as follows: 1) excitation wavelength: 785 nm; 2) laser power: medium; 3) spectral wavenumber resolution: max. 10 cm^−1^; 4) detection spectral range: 400–2300 cm^−1^; 5) detector type: linear charge-coupled device (CCD) array. The scan time for each spectrum was 0.47 s. All the output data were in plain text format. Before spectral acquisition, wavenumber calibrations were conducted using the Raman shift at 520 cm^−1^ as the reference peak on the silicon wafer, while the detector dark current was subtracted at the same integration time. Raw SERS spectra data for all the sputum samples and *Mtb* strains were available upon request.

### 2.4 Average SERS spectra and characteristic peaks

During SERS spectral analysis, average SERS spectrum for each study group was generated by calculating the average Raman intensity at each Raman shift in the range of 402 to 2298 cm^−1^. All the averaged Raman spectra were pre-processed using LabSpec 6 (HORIBA Scientific, Japan), including smoothing, denoising, baseline correction and normalization. Characteristic peaks of each average SERS spectrum were then identified. Specific processes were performed as follows: 1) *Smoothing* function was first used to smooth and denoise the spectrum with settings of *Degree* = 4, *Size* = 5, and *Height* = 50; 2) *Baseline Correction* was conducted through the following parameters: *Type* = Polynom, *Degree* = 6, *Attach* = No, while *Baseline Fitting* was done via *Auto* function; 3) *GaussLoren* function was used to find characteristic peaks, and the parameters were set to *Level* = 13% and *Size* = 19 while other parameters were set to default; 4) *Normalization* function was used in default settings to normalize all the SERS spectra in order to compare the average SERS spectral curves for different sample groups; 5) *Search* function was finally used to identify characteristic peaks. Characteristic peaks for each average SERS spectrum were labelled in vertical black arrows at corresponding Raman shifts. The software Origin (OriginLab, USA) was used to generate the 20% standard error band for each average SERS spectrum. The width of the standard error band reflected the reproducibility of each SERS spectrum.

### 2.5 Computational analysis of SERS spectra

#### 2.5.1 SERS spectral preprocess

The signal intensity of Raman spectra is generally weak, which is intrinsically affected by many factors such as Raman light source (power, wavelength, spot size), spectrometer (resolution, integration time), sample placement (laser beam focus spot), and sample vessel material, etc., resulting in spectral noises that were mixed with Raman signals, affecting the analytical effects. In order to improve the accuracy of computational analysis via machine learning algorithms, all the SERS spectra were pre-processed through normalization and smoothing.

##### 2.5.1.1 Normalization

Due to the high dimensionality of Raman spectral data, in order to avoid the influences of biased values of Raman intensities among SERS spectra, all data were first preprocessed by data normalization. In particular, normalization is a procedure of removing unit limitations from data and converting data into non-scalar values so that data with different units or magnitudes can be compared and weighted [23]. In addition, normalization can also accelerate model fitting process, improve computational efficiency, and enhance model learning accuracy [24]. In this study, all the data were normalized in each dimension through uniformly mapping the data to [0, 1] interval by using formula 1 below:

(1) $$x=\frac{x-Min}{\mathrm{Max}-Min}$$

##### 2.5.1.2 Spectral smoothing and denoising

In order to understand the impact of noises on Raman spectra caused by dark current and other systematic factors, we compared the performance of machine learning algorithms on SERS spectral data before and after smoothing and denoising SERS spectra via filtering algorithms in terms of the improvement of signal-to-noise ratio (SNR). A total of five filtering algorithms were included, which were Gaussian Filter (GF), Median Filter (MF), Wavelet Transform (WT), Median Average (MA), and Savitzky-Golay (SG). The effects of different filtering algorithms in reducing Raman spectral noise interference were compared, and the best filtering algorithm for SERS spectra was selected for further analysis. The results were evaluated using two indicators: SNR and Root Mean Square Error (RMSE). In particular, as a main technical indicator, SNR was normally used to measure the reliability of signal quality. The SNR formula used in this study was shown below:

(2) $$\mathrm{SNR}=10*Log_{10}\left(\frac{P_{S}}{P_{N}}\right)$$

*P_S_* represented the raw Raman spectral signal peak, and *P_N_* represented the Raman spectral signal peak after denoising. In order to further quantify the degree of differences between the raw spectral data and the data after denoising, RMSE was also introduced:

(3) $$\mathrm{RMSE}=\sqrt{P_{S}}$$

The specific parameters of the five smoothing and denoising algorithms were set as follows: 1) MA: Segment size = 3; 2) GF: Segment size = 3, standard deviation = 2; 3) MF: Segment size = 3; 4) SG: Polynomial order = 2, Number of left/right side points = 3. As for the WT algorithm, the situation was a bit complicated. In this study, we used soft threshold, hard threshold and fixed threshold to compare the SNR and RSME values, respectively, and then selected the best wavelet threshold denoising method. After comparison, the parameters were set as: tptr = sqtwolog, Sorh = s, soft threshold, Scal = sln, adjusted according to the noise level estimation of the first-layer wavelet decomposition, lev = 3, and wname = db4.

#### 2.5.2 Supervised machine learning analysis

After normalization, smoothing and denoising, four deep learning algorithm models, that is, CNN (Convolutional Neural Network), GRU (Gate Recurrent Units), LSTM (Long Short-Term Memory), and MLP (Multilayer Perceptron), together with two classic machine learning algorithms Random Forest (RF) and Support-Vector Machine (SVM), were recruited to analyze the SERS spectra computationally. During machine learning analyses, spectral data were divided into training set, validation set and test set in a ratio of 6:2:2. The SERS spectral data were analyzed in three aspects as previously defined: 1) smear-positive and smear-negative sputum samples; 2) pulmonary and extrapulmonary *Mtb* strains; and 3) drug-sensitive and drug-resistant *Mtb* strains. It should be noted that when dealing with the antibiotic profiling of *Mtb* strains, the labels in the data set were converted to One-hot Encoding form due to the presence of four subsets of SERS spectra. As for the classification of pulmonary/extrapulmonary *Mtb* strains and smear-negative/positive sputum samples, no transformation of the dataset labels was required due to their intrinsic binary classification nature.

**Fig. 1:**
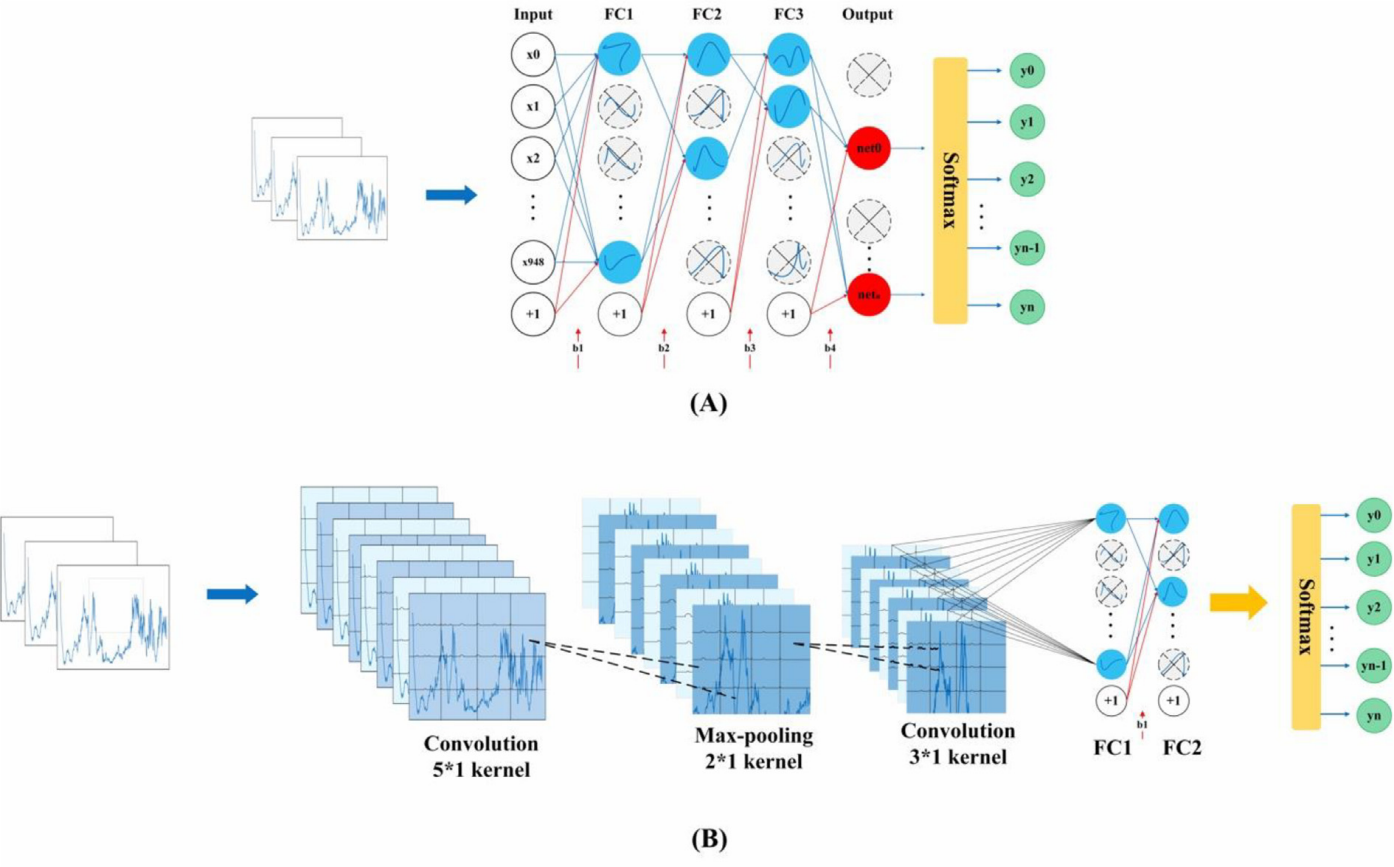
Schematic illustration of the frameworks of deep learning models of CNN and MPL. (A) MLP network structure including the input layer, the hidden layer and the output layer. Different layers of the MLP network are fully connected. (B) CNN network structure composing of input layer, convolution layer, pooling layer, fully connected layer and output layer. It can be seen that the fully connected layer of CNN is similar to that of MLP. Therefore, MLP is a special CNN network structure. When the data arrives at the fully connected layer, a multi-class neural network is performed through the Softmax activation function to obtain the final output.

**Fig. 2:**
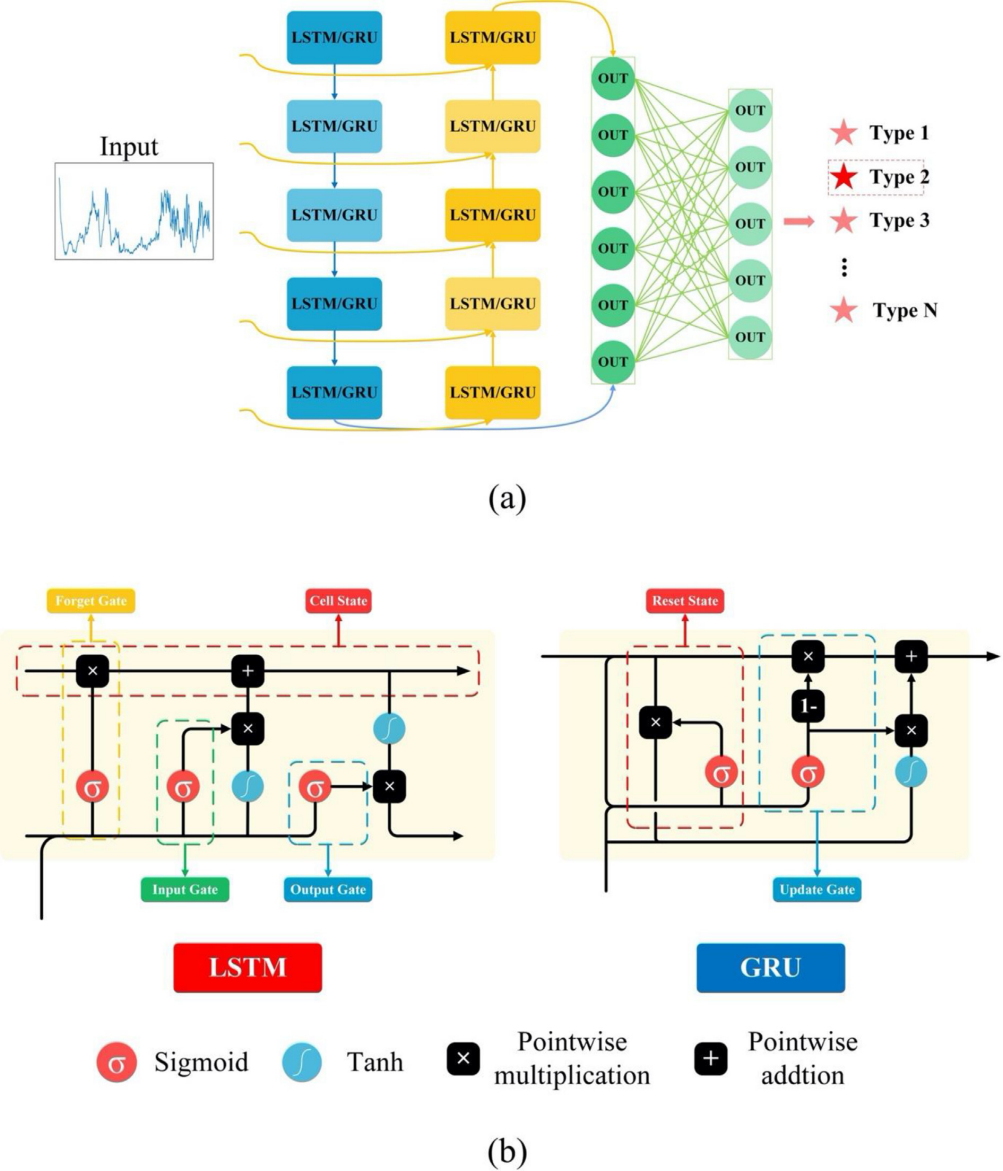
Schematic illustration of LSTM and GRU framework structures. Both LSTM and GRU can be considered as a special RNN network, where GRU is a variant of LSTM. (A) The general framework structure for LSTM and GRU mainly consisting of input layer, hidden layer (LSTM or GRU layer), dropout layer and output layer. (B) Differences between LSTM and GRU frameworks. Left half is the internal structure interaction diagram of LSTM, which includes three gate structures: Forget Gate, Input Gate and Output Gate. A Cell State gate is also included for protection and control. The GRU in the right half, as a variant of LSTM, combines the Forget Gate and the Input Gate into a single Update Gate, which also includes a Reset Gate. Although there are differences in the internal structure of LSTM and GRU, both structures can effectively prevent the phenomenon of gradient dispersion during data analysis.

In general, the deep learning models mainly have two frameworks: CNN and Recurrent Neural Networks (RNN). MLP is a special CNN network structure, which is composed of fully connected layers (also known as dense layers). Schematic illustration of the structures of the two deep learning frameworks of MLP and CNN were shown in Fig. 1. As for the LSTM model, it inherits most of the features of RNN and solves the problem of vanishing gradients in the RNN model. GRU is a simplified LSTM model, which has only two gates, that is, the update gate and the reset gate. Since GRU has fewer parameters and faster convergence, it speeds up the iterative process during model training. The illustrative structures of the two deep learning frameworks of GRU and LSTM were shown in Fig. 2. The specific structure of all the machine learning models were described below.

In specificity, CNN included one input layer, four convolutional layers and two max-pooling layers, each two convolutional layers followed by a max-pooling layer, a fully connected layer and an output layer. The *softmax* activation function was used when classifying drug-resistant/sensitive *Mtb* strains, while the *sigmoid* activation function was used when analyzing pulmonary/extrapulmonary *Mtb* strains and smear-positive/negative sputum samples. The activation function of the convolutional layer was set to “relu” while the convolution kernel sizes were 5*1 (*softmax* activation function) and 3*1 (*sigmoid* activation function), respectively (Fig. 1**A**). MLP consisted of one input layer, four fully connected layers and one output layer; in addition, *Dropout* layer was used to prevent overfitting and improve the generalization ability of the model (Fig. 1**B**). For both LSTM and GRU models, the same model structure was used, which consisted of two RNN layers, two *Dropout* layers and a fully connected layer (Fig. 2). During the model training process, the test set was completely independent of the training set and the validation set. The model trained on the training set and the validation set was tested using the test set to avoid overfitting. For the two classical learning algorithms RF and SVM, before analyzing the test set data, grid search algorithm *GridSearch* was used to test the two models on the training and validation data set to obtain the best parameters. The optimal parameters were passed into the model to classify and predict the test set data (**Supplementary** Table S4-S6).

This study also measured the generalization ability of the six models by calling the evaluation metrics from the scikit learn library https://scikit-learn.org in order to compare their prediction performances. The most common evaluation index was Accuracy (ACC), that is, the proportion of accurately classified samples. In order to verify the reliability of the ACC score, we used FiveFold Cross-Validation (CV) for the analysis. FiveFold CV can also eliminate the disadvantages caused by data imbalance. For example, during the prediction of drug-resistant and drug-sensitive *Mtb* strains, there were 1410 SERS spectra from 47 R-/H- *Mtb* strains, while only 270 SERS spectra from 9 R+/H- *Mtb* strains. Such a biased distribution of spectral numbers may cause larger samples to have a negative impact on smaller samples, resulting in poor model prediction performance. In addition, we also use Precision (P), Recall (R) and F1-score as evaluation metrics, where F1-score is equivalent to the harmonic mean of P and R. The larger the F1, the better the model performance.

#### 2.5.3 ROC curves

To further verify the model performance, we also calculated the ROC curve for each of all the six supervised machine learning models, which could easily find out the ability of a classifier model to identify samples at a certain threshold. Lines with different colors were used to represent the ROC curves of different models. The closer the ROC curve is to the upper left corner, the higher the sensitivity, the lower the false positive rate, and the better the diagnostic performance of a model. It can also be seen that the point on the ROC curve closest to the upper left corner on the ROC curve has the largest sum of sensitivity and specificity. This point is also called the optimal critical point [25]. Finally, the area under curve (AUC) value ​​under the ROC curve of each model was calculated. The large the AUC value, the better the model performance.

#### 2.5.4 Confusion matrix

**Fig. 3:**
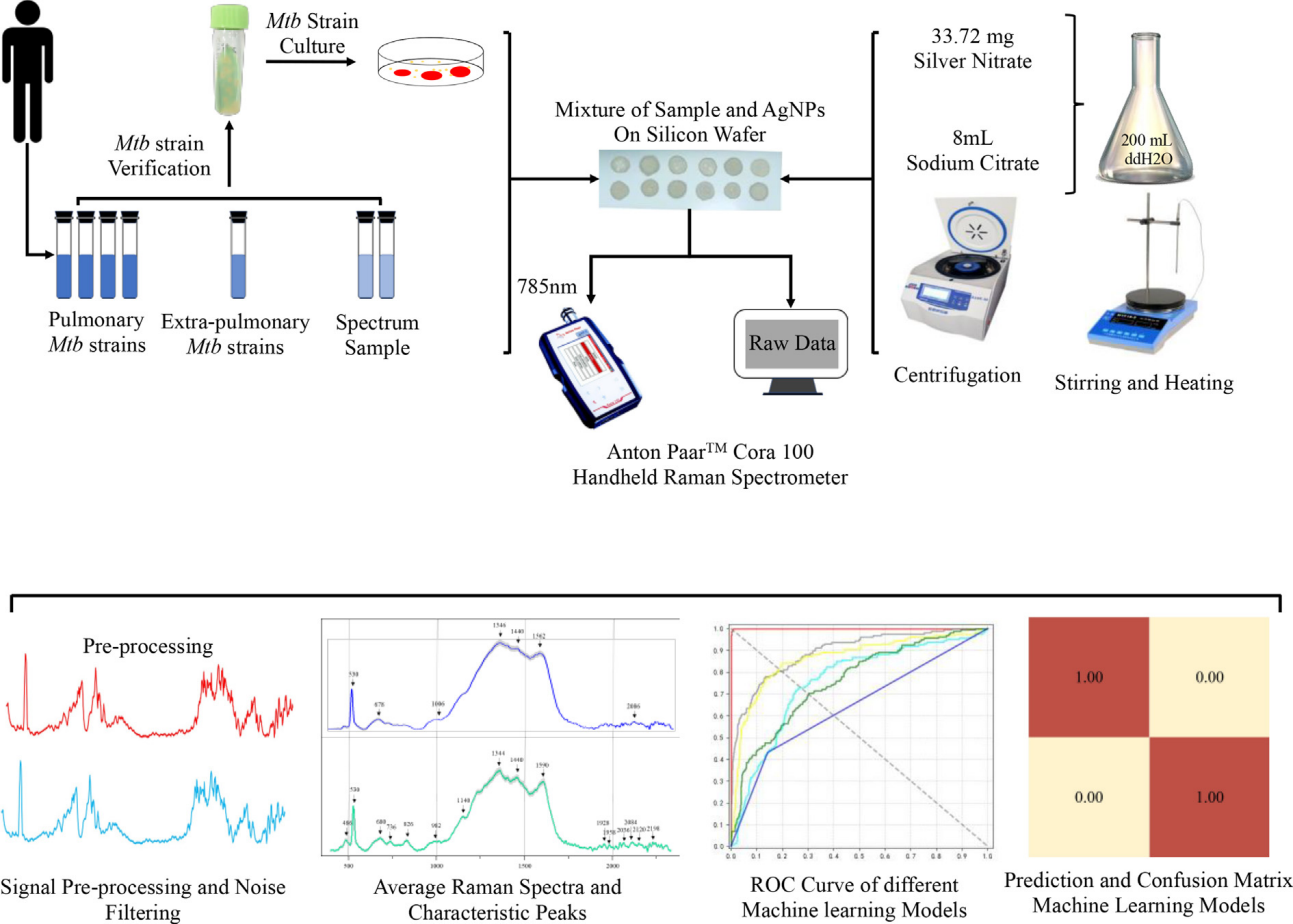
Schematic summary of the SERS-based diagnostics of *Mtb* strains and clinical sputum samples. In particular, *Mtb* strain or sputum sample was first mixed with silver nanoparticles (AgNPs) and then dried on silicon wafer. SERS fingerprinting data in the smearing dot area were then produced. Raw SERS spectra were pre-processed, averaged, and then analyzed through a series of computational steps.

Confusion matrix is ​​a table for summarizing classification and prediction results during machine learning analysis. The records in the data set are summarized in matrix form according to the two criteria of the real category and the classification judgment made by the classification model [25]. Each column in the confusion matrix represents the predicted class, and each row represents the true class of the data. Through the analysis of the samples by a machine learning model, it is able to predict which data is positive or negative. Four indicators can be obtained through the analysis: True Positive (TP), False Negative (FN), False Positive (FP), and True Negative (TN). In this study, we trained six machine learning models, and by comparing the performance of the six models, CNN was identified as the best prediction model, the confusion matrix of which was calculated for further analysis. A complete procedure of the experimental and computational studies was summarized in the flow chart below (Fig. 3).

## 3 Results and discussion

Surface enhanced Raman spectroscopy has potential for rapid and accurate diagnosis of various infectious diseases when combined with machine learning algorithms, which shows great advantages in the turnaround time, operation procedures, and testing costs [21]. Previously, SERS technique has been widely applied to detect many infection-causing bacterial pathogens, such as *Escherichia coli*, *Staphylococcus aureus* and *Pseudomonas aeruginosa*, etc.; these studies normally focused on analyzing SERS spectra and corresponding characteristic peaks via statistical methods such as partial least square-discriminant analysis (PSL-DA), principal component analysis (PCA), and hierarchical cluster analysis (HCA), etc. [26], [27], [28], [29]. However, due to the complexity of the SERS spectral data, these classical statistical methods are insufficient for data analysis and pattern recognition, which requires the assistance of advanced computational algorithms such as machine learning methods [21], [30]. In fact, a variety of studies have already applied machine learning algorithms on SERS spectroscopy for the rapid detection of bacterial pathogens, which include but not limited to support vector machine (SVM) and random forest (RF), etc. [31], [32]. Recently, deep learning algorithms were also introduced into the field for the rapid analysis of low SNR and one-dimensional SERS spectra. Ho et al. conducted the pioneering study in which the state-of-the-art CNN technique was applied to 60,000 SERS spectra for rapid identification of 30 common bacterial pathogens with the accuracy of 82% [33]. A series of studies then compared the prediction accuracies of various machine learning algorithms including deep learning algorithms in different types of bacterial pathogens [22], [30], [34]. In specificity, Tang et al. performed the comparative analysis of ten machine learning algorithms on 2752 SERS spectra of nine *Staphylococcus* species, which confirmed that the convolutional neural network (CNN) algorithm was the best model with an accuracy of 98.21% [22]. In another study, Tang et al. compared eight machine learning algorithms on the SERS spectra of 15 different bacterial pathogens, which also showed that the deep learning algorithm CNN achieved the best prediction accuracy at 99.86% [30]. Therefore, combination of SERS spectrometry with deep learning algorithm provides an advanced method with sufficient accuracy in identifying bacterial species that holds the application potential in clinical settings.

### 3.1 Discrimination of smear-negative and smear-positive sputum samples

**Fig. 4:**
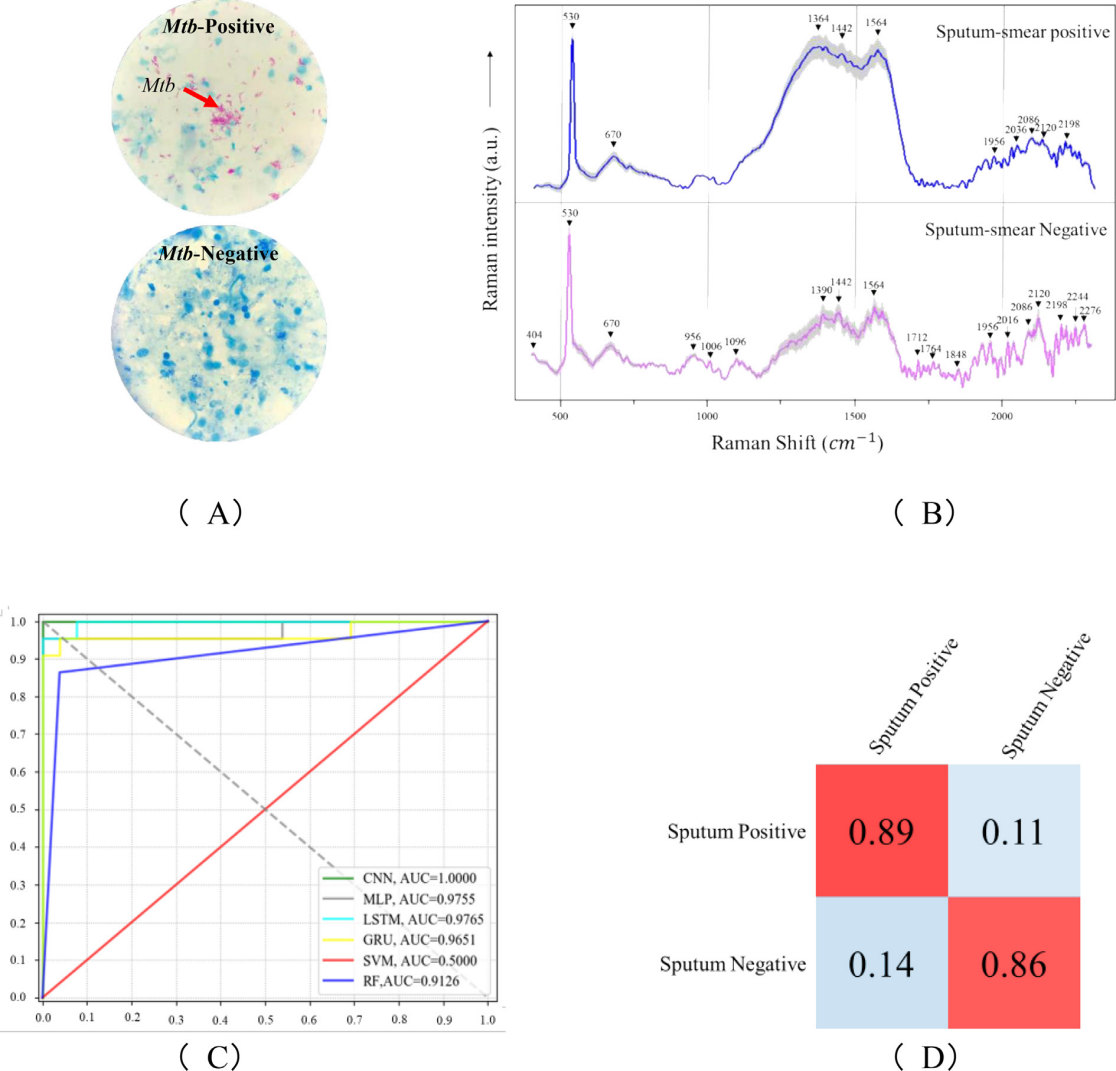
Experimental and computational analyses of smear-positive (n = 4) and -negative (n = 4) sputum samples. (A) Acid-fast bacilli testing of sputum samples. Red arrow: *Mycobacterium tuberculosis* cells in pink color. Blue areas are sputum and other bacterial cells. (B) Averaged surface enhanced Raman spectra of smear-positive and -negative sputum samples with computationally identified characteristic peaks labelled in black arrows and numbered with corresponding Raman shifts. Shadow region for each averaged spectrum represented 20% of standard error of Raman shift. (C) Comparison of receiver operating characteristic (ROC) curves used for the evaluation of the performance of 6 supervised machine learning algorithms. (D) Confusion matrix of fivefold cross-validated identification of smear-positive and -negative sputum samples via CNN model. (For interpretation of the references to color in this figure legend, the reader is referred to the web version of this article.)

In clinical laboratories, sputum is one of the most common specimens for the diagnosis of pulmonary infection with *Mycobacterium tuberculosis*, while the AFB smear test of sputum samples is frequently used to diagnose an active tuberculosis infection [35]. However, it has been suggested that sputum smear microscopy is insensitive, laborious, and time-consuming [36]. Therefore, it would be convenient if the simpler Raman spectroscopy method could be used to discriminate the smear-negative and smear-positive sputum samples directly. In this study, we first used AFB method to test the eight clinically collected samples (Fig. 4**A**), which experimentally divided the samples into *Mtb*-positive group (n = 4) and *Mtb*-negative group (n = 4). During sputum detection via SERS spectroscopy, 30 spectra were obtained for each sample and a total of 120 spectra were generated for each group, which were then combined together to calculate the averaged SERS spectrum in order to reduce the systematic errors via technical repeats (Fig. 4**B**). According to the averaged SERS spectra, comparatively high reproducibility was achieved as indicated by the thin error bands, which suggested the good applicability of handheld Raman spectrometer in the detection of clinical samples. For the averaged Raman spectrum of each sputum group, multiple characteristic peaks were identified as labelled in black arrows in Fig. 4**B**.

Some of the spectral peaks were assigned to known metabolites, though the identities of metabolites could only be speculated due to the complexity of Raman spectra [22]. Previously, many studies showed that distinctive differences were normally observed in SERS spectral data in the Raman shift region of 500–1800 cm^−1^ [22], [37], [38]. In addition, the characteristic peaks out of 1800 cm^−1^ corresponding to metabolites were rarely reported. Therefore, in this study, we mainly focused on the characteristic peaks identified in the region of 500–1800 cm^−1^ as shown in Table 1, though the range of the Raman shifts automatically generated by the handheld Raman spectrometer ranged from 402 to 2298 cm^−1^.

**Table 1:** Band assignments of characteristic peaks to potential metabolites in SERS spectra of smear-positive and -negative sputum samples.

Raman Shift (cm^−1^)	Band Assignment	Refs.
530	Silicon substrate	[39]
670	COO-bending in tyrosine, Guanine vibration	[39], [40], [41], [42]
956	C—N stretching, C=C deformation	[39], [40], [41]
1006	ν(C—C—O)	[43]
1096	Stretching of O—P—O^−^, C—O—C stretch from glycosidic link	[44]
1364	V (COO–), δ (C—H)	[45]
1390	COO-stretching	[42]
1442	δ (CH2)	[45]
1564	(C=C) stretch	[43]
1712	Amide I	[42]
2120/2198/2244/2276	C≡C	[46]

*Note: characteristic peaks that were shown in Fig. 4**B** but not listed in the table were those that could not be identified in the literature.

As seen in Fig. 4**B**, characteristic peaks in the SERS spectra (500–1800 cm-^1^) of smear-positive and -negative sputum samples were largely similar, and it was rather difficult to recognize the individual contributions of single peaks. Therefore, it is more accurate and efficient to use advanced statistical methods such as machine learning models to analyse SERS spectra overall. In this study, we compared six supervised machine learning algorithms in terms of their capacities in the analysis of SERS spectra for sputum samples through the parameters of ACC, Precision, Recall, F1 Score and FiveFold CV (Table 2). According to the result, it was revealed that all the deep learning algorithms (ACC_CNN_ = 95.67%, ACC_MLP_ = 91.92%, ACC_LSTM_ = 94.25% and ACC_GRU_ = 93.75%) performed better than the classical machine learning algorithms (ACC_SVM_ = 87.50% and ACC_RF_ = 89.58%) in terms of prediction accuracy, among which CNN had the best prediction accuracy at 95.67%. As for the fivefold cross validation, the deep learning algorithm GRU performed the best with accuracy at 95.50%.

**Table 2:** Comparison of 6 machine learning algorithms in terms of their capacities in the analysis of SERS spectral data generated from smear-positive and -negative sputum samples.

ML Algorithms	ACC	Precision	Recall	F1-Score	FiveFold CV
CNN	95.67%	94.00%	96.50%	96.37%	94.32%
MLP	91.92%	94.40%	95.45%	95.21%	93.45%
LSTM	94.25%	91.25%	91.25%	91.24%	92.67%
GRU	93.75%	93.75%	95.42%	93.73%	95.50%
SVM	87.50%	87.50%	87.41%	87.50%	91.33%
RF	89.58%	89.58%	88.99%	89.49%	93.28%

In addition, the receiver operating characteristic (ROC) curve is a graphical demonstration of true-positives and false-positives across a range of cut-offs and is normally used to assess the sensitivity and specificity of machine learning algorithms across a range of values, it was calculated in this study to compare the capacities of six machine learning algorithms in sputum SERS analysis. In addition, since the area under the ROC curves (AUCs) could be used to quantify the overall accuracies in distinguishing smear-positive and -negative samples, it was also calculated (Fig. 4**C**). The results clearly showed that deep learning algorithms had the significantly better performances than classical machine learning algorithms with CNN (AUC = 1.0) and MLP (AUC = 0.9755) as the top two algorithms. Since confusion matrix describes the classification results in terms of true class and predicted class, we also calculated it for CNN analysis, according to which smear-positive sputum samples could be correctly predicted at 89% while smear-negative sputum samples were correctly predicted at 86%.

### 3.2 Prediction of pulmonary and extra-pulmonary *Mtb* strains

**Fig. 5:**
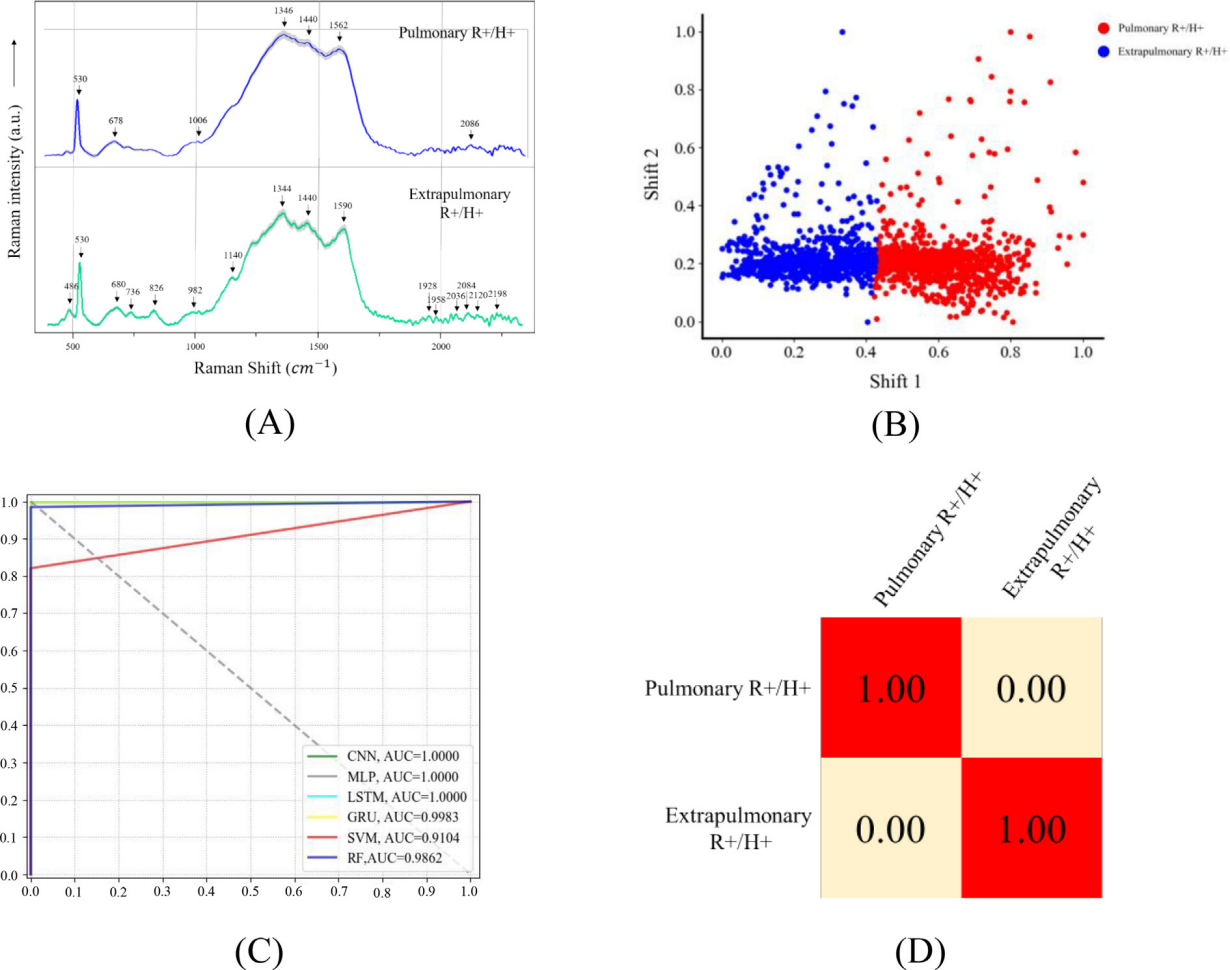
Experimental and computational analyses of pulmonary (n = 47) and extra-pulmonary *Mtb* strains (n = 11). (A) Averaged surface enhanced Raman spectra of pulmonary and extra-pulmonary *Mtb* strains with computationally identified characteristic peaks labelled in black arrows and numbered with corresponding Raman shifts. Shadow region for each averaged spectrum represented 20% of standard error of Raman shift. (B) Clustering analysis of pulmonary and extra-pulmonary *Mtb* strains via K-means algorithm. Red dots: pulmonary *Mtb* strains. Blue dots: extra-pulmonary *Mtb* strains. (C) Comparison of receiver operating characteristic (ROC) curves used for the evaluation of the performance of 6 supervised machine learning algorithms. (D) Confusion matrix of fivefold cross-validated identification of pulmonary and extra-pulmonary *Mtb* strains via CNN model. (For interpretation of the references to color in this figure legend, the reader is referred to the web version of this article.)

Although *M. tuberculosis* is primarily a respiratory pathogen and mainly targets lung for infection, there are approximately 15% of all TB infections occurring at extra-pulmonary sites, making diagnosis and treatment of tuberculosis complicated in clinical settings [47]. Therefore, it would be beneficial to quickly identify the presence of *Mtb* strains in extrapulmonary sites or discriminate extrapulmonary *Mtb* strains from pulmonary *Mtb* strains. Previously, metabolomic analysis revealed that *Mtb* could rewire their metabolic network during adaptation to different stresses [48], which also indicated that *Mtb* strains isolated from different sites could be metabolically diverse. However, few studies explored the metabolic differences of pulmonary and extrapulmonary *Mtb* strains in vivo and in vitro. Since Raman spectra are able to reveal metabolic fingerprintings for tested samples [49], we recruited the SERS technique to discriminate pulmonary and extrapulmonary *Mtb* strains with the assistance of computational methods in this study. In particular, 47 pulmonary *Mtb* strains (R+/H + ) and 11 extrapulmonary *Mtb* strains (R+/H + ) were investigated in this study. For each of *Mtb* strains, 30 spectra were collected, leading to 1441 spectra for pulmonary *Mtb* and 330 spectra for extra-pulmonary *Mtb*. The averaged SERS spectra for pulmonary and extra-pulmonary *Mtb* strain were present in Fig. 5**A** with characteristic peaks indicated by black arrows. It is noteworthy that, in order to avoid the influences of antibiotic profiles, all the *Mtb* strains used during the analysis were resistant to both isoniazid and rifampicin. For most of the characteristic peaks, their corresponding metabolites could be identified according to literature, while it is difficult for the rest to be mapped to particular metabolites. Band assignments of characteristic peaks to potential metabolites in the two SERS spectra were present in Table 3. However, since it was insufficient to describe the Raman spectra simply based on the characteristic peaks, more advanced computational methods were needed to characterize the whole spectra in order to achieve the classification and prediction of *Mtb* strains in pulmonary and extra-pulmonary samples.

**Table 3:** Band assignments of characteristic peaks to potential metabolites in SERS spectra of pulmonary and extra-pulmonary *Mycobacterium tuberculosis* strains.

Raman Shift (cm^−1^)	Band Assignment	Refs.
486	C—O—C ring deformation	[50]
530	Silicon substrate	[39]
678/680	COO– bending in tyrosine, guanine vibration	[39], [40], [41], [42]
736	Adenine	[41]
826	Phosphodiester	[51]
982/1006	ν(C—C—O)	[43]
1140	C—C stretching	[52]
1344/1346	V (COO–), δ (C—H)	[45]
1440	δ (CH2)	[45]
1562	(C=C) stretching	[43]
1590	CC str + NH2 bend, Phenylalanine, Amide II	[44], [53]
2120/2198	C≡C	[46]

In order to understand the intrinsic differences between the SERS spectra of pulmonary and extra-pulmonary *Mtb* strains, we first recruited unsupervised machine learning algorithm K-means for the clustering of all the spectral data (Fig. 5**B**), according to which the two types of *Mtb* strains were grouped into two different clusters with the Rand Index (RI) score reaching to 67.92%. In specificity, RI aims to calculate the similarity between the two clusterings and counting samples that are designated to the same or different clusters in the predicted and true clusterings. In addition, RI has the value between 0 and 1, where 0 means that the two data clusters do not agree on any pair of points and 1 suggesting that the data clusterings are exactly the same. Since the RI score in this study is only 67.92%, it suggested that the unsupervised clustering analysis was not good enough to separate the two groups of *Mtb* strains. Therefore, supervised machine learning algorithms were then applied to the spectral data for further analysis.

A total of six supervised machine learning algorithms in terms of their capacities in discriminating SERS spectra of pulmonary and extra-pulmonary *Mtb* strains. The parameters that were used to assess the prediction included ACC, Precision, Recall, F1 Score and FiveFold CV, which were present in Table 4. According to the result, it was revealed that all the machine learning algorithms showed very high prediction accuracy, among which CNN, MLP and LSTM had the best prediction accuracy. As for the fivefold cross validation, the deep learning algorithm CNN performed the best with accuracy at 99.86%. Moreover, ROC curves were drawn while the areas under the ROC curves (AUCs) were also calculated (Fig. 5**C**). The results suggested that the performance of all the deep learning algorithms was better than classical machine learning algorithms. In addition, confusion matrix was also present for CNN model, according to which both pulmonary and extra-pulmonary *Mtb* strains could be correctly predicted at 100% accuracy (Fig. 5**D**).

**Table 4:** Comparison of six machine learning algorithms in terms of their capacities in the analysis of SERS spectral data generated from pulmonary and extra-pulmonary *Mtb* strains.

ML Algorithms	ACC	Precision	Recall	F1-Score	FiveFold CV
CNN	100.00%	100.00%	100.00%	100.00%	99.86%
MLP	100.00%	100.00%	100.00%	100.00%	99.01%
LSTM	100.00%	100.00%	100.00%	100.00%	99.16%
GRU	98.85%	98.85%	97.42%	98.34%	91.44%
SVM	99.36%	100.00%	100.00%	100.00%	99.93%
RF	98.14%	98.14%	97.76%	98.13%	98.57%

### 3.3 Identification of drug-sensitive and drug-resistant *Mtb* strains

**Fig. 6:**
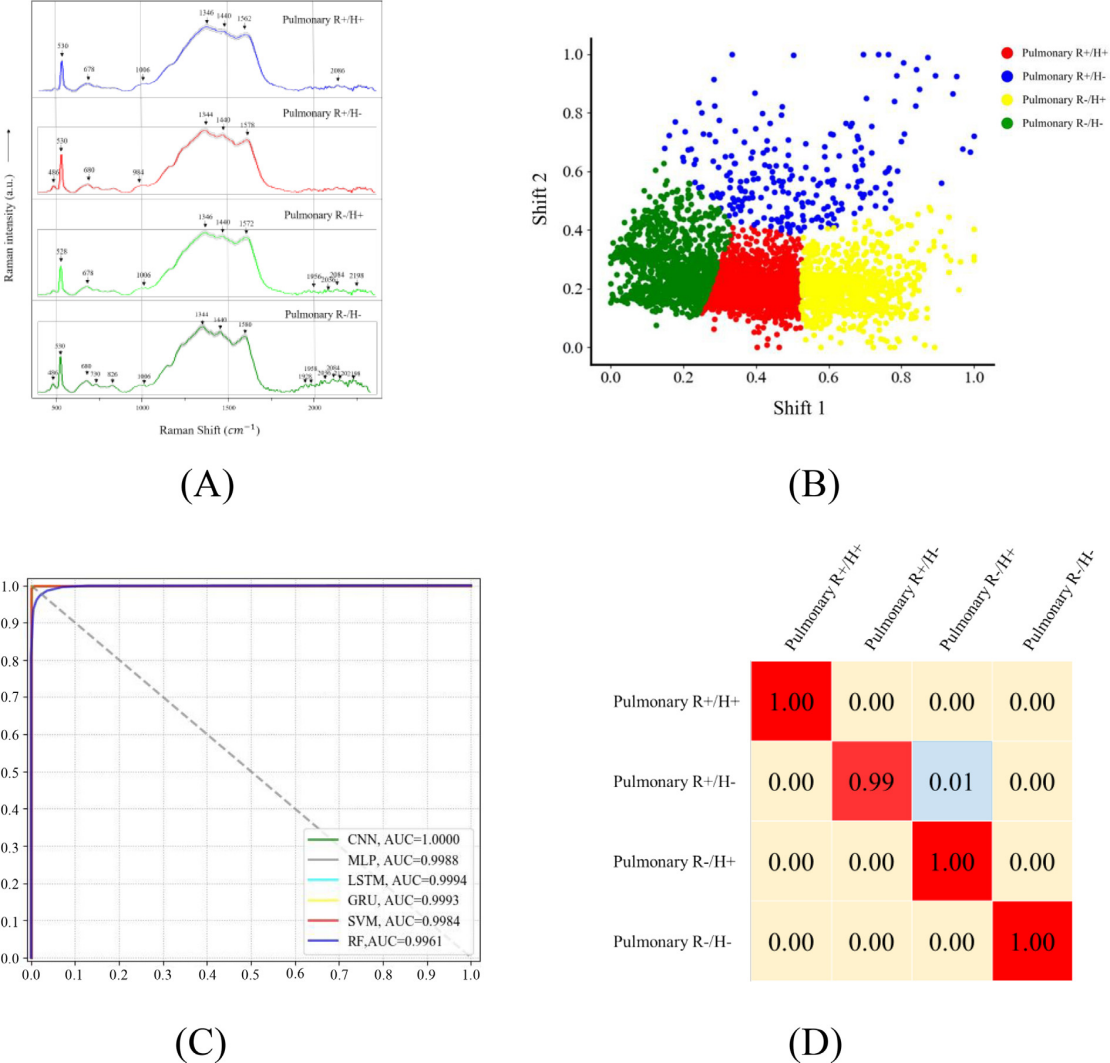
Experimental and computational analyses of four groups of drug-resistant and drug-sensitive *Mtb* strains. (A) Averaged surface enhanced Raman spectra of four groups of drug-resistant and -sensitive *Mtb* strains with computationally identified characteristic peaks labelled in black arrows and numbered with corresponding Raman shifts. Shadow region for each averaged spectrum represented 20% of standard error of Raman shift. (B) Clustering analysis of four groups of drug-resistant *Mtb* strains via K-means algorithm. Red dots: R+/H +. Blue dots: R+/H-. Yellow dots: R-/H +. Green dots: R-/H-. (C) Comparison of receiver operating characteristic (ROC) curves used for the evaluation of the performance of 6 supervised machine learning algorithms. (D) Confusion matrix of fivefold cross-validated identification of the four groups of *Mtb* strains with different drug-resistant profiles via CNN model. (For interpretation of the references to color in this figure legend, the reader is referred to the web version of this article.)

*Mycobacterium tuberculosis* is intrinsically resistant to a variety of antibiotics, which makes it rather difficult for its treatment and poses significant challenges and public risks to tuberculosis control programs [13]. In clinical settings, multidrug-resistant *Mtb* strains are defined as those that are resistant to at least two most commonly used tuberculosis drugs, isoniazid and rifampicin [54]. However, current methods for profiling the drug resistance of *Mtb* strains are either time-consuming or technically challenging, especially in LMICs [55]. Therefore, in this study, we investigated the applicability of the short-turnaround-time, non-invasive and label-free SERS technique in predicting the profiles of *Mtb* strains with the assistance of computational methods. A total of four groups of *Mtb* strains with different drug-resistant profiles were explored through SERS analysis, which were R+/H+ (n = 47), R+/H- (n = 9), R-/H+ (n = 20) and R-/H- (n = 47). Here, n represents the number of sample strains, and a total of 30 spectra were measured for each sample strain. The averaged SERS spectra for the four groups of *Mtb* strains were provided, in which characteristic peaks were indicated by black arrows and marked with specific Raman shifts (Fig. 6**A**).

The metabolites corresponding to the characteristic peaks were sourced from literature and listed in Table 5. Through the comparison of the averaged Raman spectra and also the characteristic peaks, it could be seen that the differences between the four groups were not significant and further analysis should be conducted to differentiate these spectra.

**Table 5:** Band assignment of characteristic peaks to potential metabolites in SERS spectra of the four groups of *Mtb* strains with different antibiotics-resistant profiles.

Raman Shift (cm^−1^)	Band Assignment	Refs.
486	C—O—C ring deformation	[50]
530	Skeletal mode	[39]
678/680	COO– bending in tyrosine, Guanine vibration	[39], [40], [41], [42]
730	Adenine	[41]
826	Phosphodiester	[51]
984/1006	ν(C—C—O)	[43]
1344/1346	ν(COO–), δ (C—H)	[45]
1440	δ (CH2)	[53]
1562	(C=C) stretching vibration	[43]
1572/1578/1580/1590	CC stretching vibration + NH2 bend, Phenylalanine, Amide II	[44], [53]
2120/2198	C≡C	[46]

Clustering analysis based on the unsupervised machine learning algorithm K-means was also conducted, aiming to separate SERS spectra into different groups based on their overall features rather than discrete characteristic peaks (Fig. 6**B**). According to the analysis, Rand Index that was used to assess the clustering effect was only 35.80%, which indicated that the algorithm could not divide the four types of *Mtb* strains into separate groups. In order to discriminate *Mtb* strains based on their antibiotics-resistant profiles, further computational analysis through supervised machine learning algorithms was then performed, the results of which revealed that all the tested algorithms could accurately predict different types of *Mtb* strains in terms of antibiotic resistance (Table 6). In addition, CNN topped all other algorithms with the highest prediction accuracy (ACC = 99.59%) and robustness (FiveFold CV = 99.59%), which was consistent with the analyses above when dealing with sputum-negative/positive samples and pulmonary/extra-pulmonary *Mtb* strains. Moreover, according to the ROC curves and the corresponding AUCs as shown in Fig. 6**C**, it was found that the performance of all the machine learning algorithms could reach very high value. In terms of the confusion matrix for CNN model, all the four types of *Mtb* strains could be correctly predicted at the accuracy of 99% and above (Fig. 6**D**). In fact, only 1% of R+/H- *Mtb* strains was mistakenly identified as R-/H + *Mtb* strains, while all other strains were correctly predicted with 100% accuracy.

**Table 6:** Comparison of six machine learning algorithms in terms of their capacities in the analysis of SERS spectral data generated from four groups of *Mtb* strains with different drug-resistant profiles.

ML Algorithms	ACC	Precision	Recall	F1-Score	FiveFold CV
CNN	99.59%	99.64%	99.61%	99.67%	99.59%
MLP	97.19%	97.22%	97.35%	97.22%	97.22%
LSTM	98.72%	98.73%	98.73%	98.73%	96.89%
GRU	98.93%	98.59%	98.34%	98.59%	95.67%
SVM	96.32%	96.32%	96.43%	96.33%	96.86%
RF	96.61%	96.61%	92.71%	96.70%	96.98%

### 3.4 Influences of SERS spectra pretreatment on machine learning analysis

Raw Raman spectral data are normally not suitable for the direct input into machine learning algorithms due to the low signal-to-noise ratio (SNR) [30]. Therefore, in order to improve the SNR of SERS spectra and enhance the main features in the data, all the SERS spectra need to be pre-processed before machine learning analysis [30], [34]. Therefore, in this study, we compared five different denoising algorithms and assessed their capacities via two scoring criteria, SNR and RMSE. After SERS spectral pre-processing, the higher the SNR, the better the denoising effect, while the smaller the RMSE, the better the spectral quality. For the specific analysis, SERS spectra from sputum-negative sample were used as an example and the result was shown in Table 7. The computational results revealed that GF algorithm had the best performance with maximum SNR value at 38.1128 and the minimum RMSE score at 13.633 × 10^−4^. Therefore, GF algorithm was applied to all the SERS spectra for denoising SERS spectra before machine learning analysis, which could also be generalized to future studies for the preprocessing of the SERS spectral data.

**Table 7:** The scores of SNR and RMSE obtained through different filtering algorithms.

Scores	GF	MF	WT	MA	SG
SNR	38.1128	28.5252	26.5230	25.9983	25.4500
RMSE (×10^−4^)	13.633	41.113	51.771	54.995	58.578

## 4 Conclusion

Due to the large number of tuberculosis infections around the world and the high public health risks globally, rapid and accurate methods for the diagnosis of *M. tuberculosis* are highly in demand. Currently, although conventional and molecular methods have been widely applied in clinical settings for tuberculosis detection, many limitations exist such as long turnaround time and complex procedures, etc., leading to significant challenges in TB treatment and prevention. Therefore, we explored the application of the rapid and simple SERS technique in the identification, discrimination, and prediction of *Mtb* strains. Although previous studies also explored the same issue, most of them used heavy benchtop Raman spectrometer for spectral collection that significantly restricted the capacity of technique in terms of its point-of-care test. In addition, through the comparison of machine learning algorithms in the analysis of SERS spectra, we also figured out the best computational methods for accurate discrimination of *Mtb* strains and *Mtb*-containing samples. Our results confirmed that SERS detection coupled with deep learning algorithms could discriminate smear-negative sputum from smear-positive sputum accurately (5-fold cross-validation accuracy = 94.32%), which held application potentials in clinical settings for fast screening of sputum samples. However, it should be emphasized that high bacterial concentrations in sputum samples are essential in obtaining qualified SERS spectra for further computational analysis. Since smear-test-positive *Mtb* sputums typically contain high densities of *Mtb* cells, the SERS technique was applicable to the discrimination of sputum samples. As for other bacterial infections, the low bacterial concentrations in sputums will potentially obscure the relative peak intensity and the baseline intensity and leads to the generation of low-quality SERS spectra, which made the technique unsuitable for other sputum sample analysis. In addition, extra-pulmonary *Mtb* strains were swiftly separated from pulmonary *Mtb* strains (5-fold cross-validation accuracy = 99.86%), which might be caused by their metabolic differences due to their adaptations to specific niches during infection. Finally, *Mtb* strains with diverse antibiotics-resistant profiles were also accurately differentiated (5-fold cross-validation accuracy = 99.59%). Taken together, in this study, we demonstrated the application potential of handheld Raman spectrometer in terms of rapid and accurate diagnosis of *Mtb* strains in clinical samples and also with different features e.g., infection sites and antibiotics-resistant profiles, through the integration of machine learning algorithms. Thus, the combination of SERS technique and computational methods are promising for rapid point-of-care diagnosis of *Mtb* infections in clinical settings, which should be further developed and evaluated in future studies.

## 5 Novelty Statement

In this study we applied the portable handheld Anton Paar CORA100 Raman spectrometer to conduct the detection of severe pathogen *Mycobacterium tuberculosis* (*Mtb*) in three clinical settings within Physical Containment Level 3 (PC3) facility: 1) smear-positive and smear-negative sputum samples; 2) pulmonary and extra-pulmonary *Mtb* strains; and 3) pulmonary *Mtb* strains with different antibiotic resistance profiles. Our study is the first systematic investigation of *Mtb* detection via the combination of SERS technique and machine learning algorithms, which not only successfully discriminated clinical sputum samples with/without *Mtb* cells, but also accurately predicted *Mtb* strains from different infection locations and having different antibiotic resistance profiles. Thus, our study supported that handheld Raman spectrometer had a high application potential in real-world settings for rapid point-of-care diagnosis of *Mtb* infections.

## Funding Statement

Liang Wang was financially supported by 10.13039/100016163National Natural Science Foundation of China [Grant No 31900022], Young Science and Technology Innovation Team of Xuzhou Medical University [Grant Number TD202001] and Jiangsu Qinglan Project [2020]. Bing Gu thanks the financial support of the 10.13039/100016163National Natural Science Foundation of China [Grant Number 81871734 and 82072380], Key R & D Program of Jiangsu Province [Grant Number BE2020646], and 10.13039/100016163Research Foundation for Advanced Talents of Guandong Provincial People’s Hospital [Grant Number KJ012021097].

## CRediT authorship contribution statement

**Liang Wang:** Methodology, Formal analysis, Investigation, Writing – original draft, Writing – review & editing, Visualization, Project administration, Funding acquisition. **Xue-Di Zhang:** Methodology, Formal analysis, Writing – original draft. **Jia-Wei Tang:** Formal analysis, Investigation, Writing – original draft, Writing – review & editing, Visualization. **Zhang-Wen Ma:** Investigation, Validation. **Muhammad Usman:** Validation, Data curation. **Qing-Hua Liu:** Investigation, Validation. **Chang-Yu Wu:** Supervision, Project administration. **Fen Li:** Conceptualization, Methodology, Resources, Supervision, Project administration. **Zuo-Bin Zhu:** Conceptualization, Methodology, Resources, Supervision, Project administration. **Bing Gu:** Conceptualization, Methodology, Resources, Supervision, Project administration, Funding acquisition.

## Declaration of Competing Interest

The authors declare that they have no known competing financial interests or personal relationships that could have appeared to influence the work reported in this paper.
