# Supplementary material for: Machine learning analysis of SERS fingerprinting for the rapid determination of Mycobacterium tuberculosis infection and drug resistance
Source: Comput Struct Biotechnol J. 2022 Sep 26;20:5364–77. doi: 10.1016/j.csbj.2022.09.031 (PMC9526180; doi:10.1016/j.csbj.2022.09.031)
Supplement: Supplementary data 1 [file mmc1.docx]

**Supplementary Table S1** Information of pulmonary *Mtb* strains and the number of SERS spectra for each *Mtb* strain.

| **Groups** | **Number of *Mtb* Strains** | **Gneder** | **Specimen type** | **Clinical Diagnosis** | **No of Spectra** | **Total Number of Spectra** |  |
| --- | --- | --- | --- | --- | --- | --- | --- |
|  |  |  |  |  |  |  |  |
| **Pulmonary R+/H+** | **47** | Female | Broncho alveolar lavage | Tuberculosis | 30 | 1410 |  |
|  |  | Male | Broncho alveolar lavage | Tuberculosis | 30 |  |  |
|  |  | Male | Sputum | Tuberculosis | 30 |  |  |
|  |  | Male | Sputum | Tuberculosis | 30 |  |  |
|  |  | Female | Broncho alveolar lavage | Tuberculosis | 30 |  |  |
|  |  | Male | Sputum | Tuberculosis | 30 |  |  |
|  |  | Male | Sputum | Tuberculosis | 30 |  |  |
|  |  | Male | Broncho alveolar lavage | Tuberculosis | 30 |  |  |
|  |  | Male | Sputum | Tuberculosis | 30 |  |  |
|  |  | Male | Sputum | Tuberculosis | 30 |  |  |
|  |  | Male | Sputum | Tuberculosis | 30 |  |  |
|  |  | Male | Sputum | Tuberculosis | 30 |  |  |
|  |  | Male | Sputum | Tuberculosis | 30 |  |  |
|  |  | Male | Sputum | Tuberculosis | 30 |  |  |
|  |  | Male | Sputum | Tuberculosis | 30 |  |  |
|  |  | Female | Broncho alveolar lavage | Tuberculosis | 30 |  |  |
|  |  | Male | Sputum | Tuberculosis | 30 |  |  |
|  |  | Male | Sputum | Tuberculosis | 30 |  |  |
|  |  | Male | Sputum | Tuberculosis | 30 |  |  |
|  |  | Male | Sputum | Tuberculosis | 30 |  |  |
|  |  | Male | Sputum | Tuberculosis | 30 |  |  |
|  |  | Male | Broncho alveolar lavage | Tuberculosis | 30 |  |  |
|  |  | Male | Sputum | Tuberculosis | 30 |  |  |
|  |  | Female | Sputum | Tuberculosis | 30 |  |  |
|  |  | Male | Sputum | Tuberculosis | 30 |  |  |
|  |  | Male | Sputum | Tuberculosis | 30 |  |  |
|  |  | Male | Sputum | Tuberculosis | 30 |  |  |
|  |  | Male | Sputum | Tuberculosis | 30 |  |  |
|  |  | Male | Sputum | Tuberculosis | 30 |  |  |
|  |  | Male | Sputum | Tuberculosis | 30 |  |  |
|  |  | Female | Sputum | Tuberculosis | 30 |  |  |
|  |  | Male | Sputum | Tuberculosis | 30 |  |  |
|  |  | Male | Sputum | Tuberculosis | 30 |  |  |
|  |  | Male | Sputum | Tuberculosis | 30 |  |  |
|  |  | Male | Sputum | Tuberculosis | 30 |  |  |
|  |  | Female | Sputum | Tuberculosis | 30 |  |  |
|  |  | Female | Broncho alveolar lavage | Tuberculosis | 30 |  |  |
|  |  | Male | Sputum | Tuberculosis | 30 |  |  |
|  |  | Male | Sputum | Tuberculosis | 30 |  |  |
|  |  | Male | Sputum | Tuberculosis | 30 |  |  |
|  |  | Female | Sputum | Tuberculosis | 30 |  |  |
|  |  | Female | Broncho alveolar lavage | Tuberculosis | 30 |  |  |
|  |  | Male | Sputum | Tuberculosis | 30 |  |  |
|  |  | Male | Sputum | Tuberculosis | 30 |  |  |
|  |  | Male | Sputum | Tuberculosis | 30 |  |  |
|  |  | Male | Sputum | Tuberculosis | 30 |  |  |
|  |  | Male | Sputum | Tuberculosis | 30 |  |  |
| **Pulmonary R-/H+** | **9** | Male | Broncho alveolar lavage | Tuberculosis | 30 | 270 |  |
|  |  | Male | Sputum | Tuberculosis | 30 |  |  |
|  |  | Female | Sputum | Tuberculosis | 30 |  |  |
|  |  | Female | Sputum | Tuberculosis | 30 |  |  |
|  |  | Male | Sputum | Tuberculosis | 30 |  |  |
|  |  | Male | Sputum | Tuberculosis | 30 |  |  |
|  |  | Male | Sputum | Tuberculosis | 30 |  |  |
|  |  | Male | Sputum | Tuberculosis | 30 |  |  |
|  |  | Male | Sputum | Tuberculosis | 30 |  |  |
| **Pulmonary R+/H-** | **20** | Male | Sputum | Tuberculosis | 30 | 600 |  |
|  |  | Male | Sputum | Tuberculosis | 30 |  |  |
|  |  | Male | Sputum | Tuberculosis | 30 |  |  |
|  |  | Female | Sputum | Tuberculosis | 30 |  |  |
|  |  | Male | Sputum | Tuberculosis | 30 |  |  |
|  |  | Male | Sputum | Tuberculosis | 30 |  |  |
|  |  | Male | Sputum | Tuberculosis | 30 |  |  |
|  |  | Male | Sputum | Tuberculosis | 30 |  |  |
|  |  | Male | Sputum | Tuberculosis | 30 |  |  |
|  |  | Male | Sputum | Tuberculosis | 30 |  |  |
|  |  | Male | Sputum | Tuberculosis | 30 |  |  |
|  |  | Male | Sputum | Tuberculosis | 30 |  |  |
|  |  | Female | Sputum | Tuberculosis | 30 |  |  |
|  |  | Female | Sputum | Tuberculosis | 30 |  |  |
|  |  | Female | Sputum | Tuberculosis | 30 |  |  |
|  |  | Female | Sputum | Tuberculosis | 30 |  |  |
|  |  | Male | Sputum | Tuberculosis | 30 |  |  |
|  |  | Female | Sputum | Tuberculosis | 30 |  |  |
|  |  | Male | Sputum | Tuberculosis | 30 |  |  |
|  |  | Male | Sputum | Tuberculosis | 30 |  |  |
| **Pulmonary R-/H-** | **47** | Male | Sputum | Tuberculosis | 30 | 1410 |  |
|  |  | Female | Sputum | Tuberculosis | 30 |  |  |
|  |  | Male | Sputum | Tuberculosis | 30 |  |  |
|  |  | Female | Sputum | Tuberculosis | 30 |  |  |
|  |  | Male | Sputum | Tuberculosis | 30 |  |  |
|  |  | Male | Sputum | Tuberculosis | 30 |  |  |
|  |  | Male | Sputum | Tuberculosis | 30 |  |  |
|  |  | Male | Sputum | Tuberculosis | 30 |  |  |
|  |  | Male | Sputum | Tuberculosis | 30 |  |  |
|  |  | Male | Sputum | Tuberculosis | 30 |  |  |
|  |  | Male | Sputum | Tuberculosis | 30 |  |  |
|  |  | Male | Sputum | Tuberculosis | 30 |  |  |
|  |  | Female | Sputum | Tuberculosis | 30 |  |  |
|  |  | Male | Sputum | Tuberculosis | 30 |  |  |
|  |  | Male | Sputum | Tuberculosis | 30 |  |  |
|  |  | Male | Sputum | Tuberculosis | 30 |  |  |
|  |  | Male | Sputum | Tuberculosis | 30 |  |  |
|  |  | Male | Sputum | Tuberculosis | 30 |  |  |
|  |  | Male | Broncho alveolar lavage | Tuberculosis | 30 |  |  |
|  |  | Male | Sputum | Tuberculosis | 30 |  |  |
|  |  | Female | Broncho alveolar lavage | Tuberculosis | 30 |  |  |
|  |  | Male | Sputum | Tuberculosis | 30 |  |  |
|  |  | Male | Sputum | Tuberculosis | 30 |  |  |
|  |  | Male | Sputum | Tuberculosis | 30 |  |  |
|  |  | Female | Sputum | Tuberculosis | 30 |  |  |
|  |  | Male | Sputum | Tuberculosis | 30 |  |  |
|  |  | Male | Sputum | Tuberculosis | 30 |  |  |
|  |  | Male | Broncho alveolar lavage | Tuberculosis | 30 |  |  |
|  |  | Male | Sputum | Tuberculosis | 30 |  |  |
|  |  | Male | Sputum | Tuberculosis | 30 |  |  |
|  |  | Male | Broncho alveolar lavage | Tuberculosis | 30 |  |  |
|  |  | Male | Sputum | Tuberculosis | 30 |  |  |
|  |  | Male | Sputum | Tuberculosis | 30 |  |  |
|  |  | Male | Sputum | Tuberculosis | 30 |  |  |
|  |  | Male | Broncho alveolar lavage | Tuberculosis | 30 |  |  |
|  |  | Male | Sputum | Tuberculosis | 30 |  |  |
|  |  | Male | Sputum | Tuberculosis | 30 |  |  |
|  |  | Male | Sputum | Tuberculosis | 30 |  |  |
|  |  | Male | Sputum | Tuberculosis | 30 |  |  |
|  |  | Female | Sputum | Tuberculosis | 30 |  |  |
|  |  | Male | Sputum | Tuberculosis | 30 |  |  |
|  |  | Female | Broncho alveolar lavage | Tuberculosis | 30 |  |  |
|  |  | Male | Sputum | Tuberculosis | 30 |  |  |
|  |  | Female | Broncho alveolar lavage | Tuberculosis | 30 |  |  |
|  |  | Female | Broncho alveolar lavage | Tuberculosis | 30 |  |  |
|  |  | Female | Sputum | Tuberculosis | 30 |  |  |
|  |  | Male | Sputum | Tuberculosis | 30 |  |  |

**Supplementary Table S2** Information of extra-pulmonary *Mtb* strains (R+/H+) and the number of SERS spectra for each *Mtb* strain.

| **Groups** | **Number of *Mtb* Strains** | **Gneder** | **Specimen type** | **Clinical Diagnosis** | **No of Spectra** | **Total Number of Spectra** |  |
| --- | --- | --- | --- | --- | --- | --- | --- |
|  |  |  |  |  |  |  |  |
| **Extra-pulmonary R+/H+** |  | Male | Pleuroperitoneal fluids | Tuberculous Pleurisy | 30 | 330 |  |
|  |  | Female | Urine | Tuberculosis | 30 |  |  |
|  | **11** | Male | Pleuroperitoneal fluids | Pleural effusion | 30 |  |  |
|  |  | Female | Pus | Tuberculosis of ankle joint | 30 |  |  |
|  |  | Male | Urine | Tuberculosis | 30 |  |  |
|  |  | Male | Pus | Lymphoid Tuberculosis | 30 |  |  |
|  |  | Male | Pus | Lymphoid Tuberculosis | 30 |  |  |
|  |  | Male | Pus | Thoracic Tuberculosis | 30 |  |  |
|  |  | Male | Pus | Thoracic Tuberculosis | 30 |  |  |
|  |  | Female | Urine | Tuberculosis | 30 |  |  |
|  |  | Male | Pus | Lymphoid Tuberculosis | 30 |  |  |

**Supplementary Table S3** Information of smear-positive and smear-negative sputum samples and the number of SERS spectra for each sputum sample.

| **Group** | **Sample Numbers** | **Specimen Type** | **Clinical Diagnosis** | **Number of Spectra** | **Total Number of Spectra** |  |
| --- | --- | --- | --- | --- | --- | --- |
|  |  |  |  |  |  |  |
| **Smear-Positive Sputum** | 4 | Sputum | Tuberculosis | 30 | 120 |  |
|  |  | Sputum | Tuberculosis | 30 |  |  |
|  |  | Sputum | Tuberculosis | 30 |  |  |
|  |  | Sputum | Tuberculosis | 30 |  |  |
| **Smear-Negative Sputum** | 4 | Sputum | - | 30 | 120 |  |
|  |  | Sputum | - | 30 |  |  |
|  |  | Sputum | - | 30 |  |  |
|  |  | Sputum | - | 30 |  |  |

**Supplementary Table S4** Optimal model parameters of *Mtb* strains with different antibiotic resistance profiles.

| **Model** | **Parameter** |
| --- | --- |
| CNN | Conv1D*6;  Maxpooling*3;  Dense*1;  activation='relu'&'Softmax';  kernel size=5*1,3*1;  loss='categorical_crossentropy';  optimizer='adam' |
| MLP | Dense*4;  Dropout*3;  activation='relu'&'Softmax';  loss='categorical_crossentropy';  optimizer='adam' |
| LSTM | LSTM*2;  Dropout*2;  Dense*1;  activation='relu'&'Softmax';  loss='categorical_crossentropy';  optimizer='adam' |
| GRU | GRU*2;  Dropout*2;  Dense*1;  activation='relu'&'Softmax';  loss='categorical_crossentropy';  optimizer='adam' |
| SVM | C': 0.1;  'gamma': 0.0001;  'cache_size':200;  'kernel':'linear';  'decision_function_shape':'ovr' |
| RF | criterion': 'entropy';  'max_depth': 8;  'n_estimators': 150;  'max_features':'auto' |

**Supplementary Table S5** Optimal model parameters of pulmonary and extrapulmonary *Mtb* strains.

| **Model** | **Parameter** |
| --- | --- |
| CNN | Conv1D*6;  Maxpooling*3;  Dense*1;  activation='relu'&Sigmoid';  kernel size=5*1,3*1;  loss='binary_crossentropy';  optimizer='adam' |
| MLP | Dense*4;  Dropout*3;  activation='relu'&'Sigmoid';  loss='binary_crossentropy';  optimizer='adam' |
| LSTM | LSTM*2;  Dropout*2;  Dense*1;  activation='relu'&'Sigmoid';  loss='binary_crossentropy';  optimizer='adam' |
| GRU | GRU*2;  Dropout*2;  Dense*1;  activation='relu'&'Sigmoid';  loss='binary_crossentropy';  optimizer='adam' |
| SVM | C': 0.1;  'gamma': 0.0001;  'cache_size':200;  'kernel':'linear';  'decision_function_shape':'ovr' |
| RF | criterion': 'entropy';  'max_depth': 3;  'n_estimators': 145;  'max_features':'auto' |

**Supplementary Table S6** Optimal model parameters of *Mtb*-negative and *Mtb*-positive sputum samples.

| **Model** | **Parameter** |
| --- | --- |
| CNN | Conv1D*6;  Maxpooling*3;  Dense*1;  activation='relu'&Sigmoid';  kernel size=5*1,3*1;  loss='binary_crossentropy';  optimizer='adam' |
| MLP | Dense*4;  Dropout*3;  activation='relu'&'Sigmoid';  loss='binary_crossentropy';  optimizer='adam' |
